# Asthma: From Phenotypes to Endotypes—Beyond Type 2 Inflammation

**DOI:** 10.1002/resp.70322

**Published:** 2026-10-07

**Authors:** Mario Fernando, Gregory G. King

**Affiliations:** ^1^ Department of Respiratory and Sleep Medicine Royal North Shore Hospital St Leonards New South Wales Australia; ^2^ Northern Clinical School, Faculty of Health and Medicine, University of Sydney Camperdown Australia; ^3^ The Woolcock Institute of Medical Research, Macquarie University Macquarie Park Australia

**Keywords:** airway remodelling, asthma, endotype, multi‐omics, personalised medicine, phenotype, T2‐high asthma, T2‐low asthma

## Abstract

Asthma is a heterogeneous syndrome featuring variable pathophysiology, clinical presentation and treatment response. This review examines a comprehensive endotype‐phenotype framework for asthma assessment with an emphasis on characteristics beyond T2 inflammatory status alone. Endo‐phenotype assessment can be performed across three domains: clinical (symptoms, exacerbations, co‐morbidities), structural and physiological, and inflammatory endotype. The clinical domain includes mucus hypersecretion, perception of airway narrowing, and obesity‐related asthma. Airway remodelling is the central, structural endo‐phenotype that defines asthma, given its relationship with fixed airflow limitation, airway hyperresponsiveness, small airway dysfunction, lung function decline, and asthma severity. However, it is the most difficult parameter to measure, and it is increasingly recognised that there is a critical need to be able to measure it longitudinally, beyond bronchial biopsy. Finally, T2‐high asthma is relatively well characterised with several effective anti‐inflammatory therapies available, including a range of biologics. In contrast, T2‐low asthma remains a heterogenous and potentially difficult to characterise endo‐phenotype, although some treatment studies have demonstrated benefit when subjects have been appropriately characterised and selected. Emerging multi‐omic approaches may enhance the detailed characterisation of patients in future. Adopting an endo‐phenotype framework allows clinicians and researchers to confront the complexity of asthma, better manage patients with a precision medicine approach and enhance mechanistic understanding, which is critical for new therapeutics to be developed for asthma.

## 1 Introduction

Asthma has a prevalence of 6.6% globally in adults but disproportionately high prevalence in Australasia, and high mortality rates in Oceania and South Asia [1]. Asthma is a highly heterogenous syndrome defined by respiratory symptoms including wheeze, chest tightness, cough or dyspnoea, airflow limitation, airway hyperresponsiveness (AHR), airway inflammation and airway remodelling. Each of these factors is highly variable, especially remodelling and inflammation. Asthma is best described by the word ‘variability’ in that every aspect of the disease is variable, from molecular mechanisms to clinical appearances.

The breakthrough of inhaled corticosteroids over 50 years ago [2], was an immense leap in asthma treatment, and particularly when combined with long acting β2 agonists (LABA), has led to lives saved and improved quality of life for millions of patients. Yet the disease burden remains high today, particularly in the Asia‐Pacific region [1], the decline in mortality seen between 1993 and 2006 [3] has ‘bottomed out’ despite most asthma deaths being considered preventable [3, 4] and the global incidence is even projected to increase between 2021 and 2050 [1].

Acknowledgement of great clinical heterogeneity has not progressed to improved understanding of the varying mechanisms that underlie this heterogeneity. The simple concept that eosinophils drive disease in a subgroup and that eosinophils indicate corticosteroid responsive asthma, which was reported in 1954 by Dr. Morrow Brown [5], has led to more targeted prescription of inhaled corticosteroids (ICS) and eosinophil–targeted biologics. In some, anti‐IL‐5 treatment has been ‘life‐changing’, leading to clinical remission. Targeting up‐stream inflammatory signals, for example, tezepelumab, affords benefit to T2‐low asthma but its greatest benefit is in T2‐high [6]. These new treatments are starting to influence research to better understand mechanisms of airway remodelling and mucus plugging [7]. Hopefully, this will continue to accelerate and find mechanisms of; fixed airflow limitation, AHR, poor treatment response in severe asthma, exacerbation propensity, adult‐onset disease, disease progression from infancy to older age, Type 2 low asthma and day‐to‐day lung function variability, to name some clinical phenotypes.

Clinicians face the challenge of managing a broad range of endo‐phenotypes (the shortened term ‘endotypes’). We define asthma endotypes as molecular pathways that can define subgroups of asthma (which may or may not appear clinically different), which can be distinguished by differential responses to targeted treatments [8]. Although asthma guidelines are useful in embedding the principle of sufficient treatment to achieve control of symptoms and prevent exacerbations (which is how most clinicians define asthma control), and for establishing policies, they have little relevance to practice at the individual patient level, given the complexity and consequent heterogeneity of underlying mechanisms.

Many medical specialties are evolving to personalised treatment models. Oncology has shown spectacular evolution driven by greater mechanistic understanding and better tumour measurement tools, for example, positron emission tomography and computerised image analysis. Such progress is yet to happen in asthma. In his 2008 editorial, Anderson [8] argued the need for clinicians and researchers to view asthma in a more in‐depth and comprehensive manner, that is, to think of endotypes or mechanisms which drive pathophysiology and lead to outward clinical manifestations. This is analogous to not all non‐small cell lung cancers being the same, with differing driving mutations which influence clinical behaviour and treatment responses. Asthma is just the same. Ten years later, the 2018 Lancet Commission on asthma [9] reflected on the same lack of focus on mechanisms to subtype asthma, to allow identification of new‐targeted treatments, and a lack of biomarkers and other tools for clinical adoption. Today, 8 years after the commission, the success of the T2 focussed biologics has proven that T2 phenotyping is critical for new treatments, but where to from here?

In this review, we suggest that even with the very limited understanding of asthma, a comprehensive assessment of an asthma patient in the clinic, which incorporates our currently limited tools available (clinical, inflammatory, physiologic and structural parameters), enables optimal, personalised management. We also suggest that clinicians and researchers must embrace the complexity that underlies asthma, and demand greater knowledge of the complex interplay between molecular, cellular, structural and physiologic processes [10], so that we can better manage patients and see progress towards the next generation of treatments.

## 2 What Is Asthma?

Our concept of asthma is shown in Figure 1. Central to asthma are changes to lung and airway structure from gross anatomical to ultrastructural levels (remodelling), which allow airways to narrow excessively (increased sensitivity) and by too much (increased maximal potential narrowing); together these describe airway hyperresponsiveness (AHR). It is not possible to make structurally normal airways hyperresponsive. Mechanical forces in the lungs and airway tissues including airway smooth muscle (ASM) allow protective mechanisms, particularly airway stretch, to limit airway narrowing [11].

**FIGURE 1:**
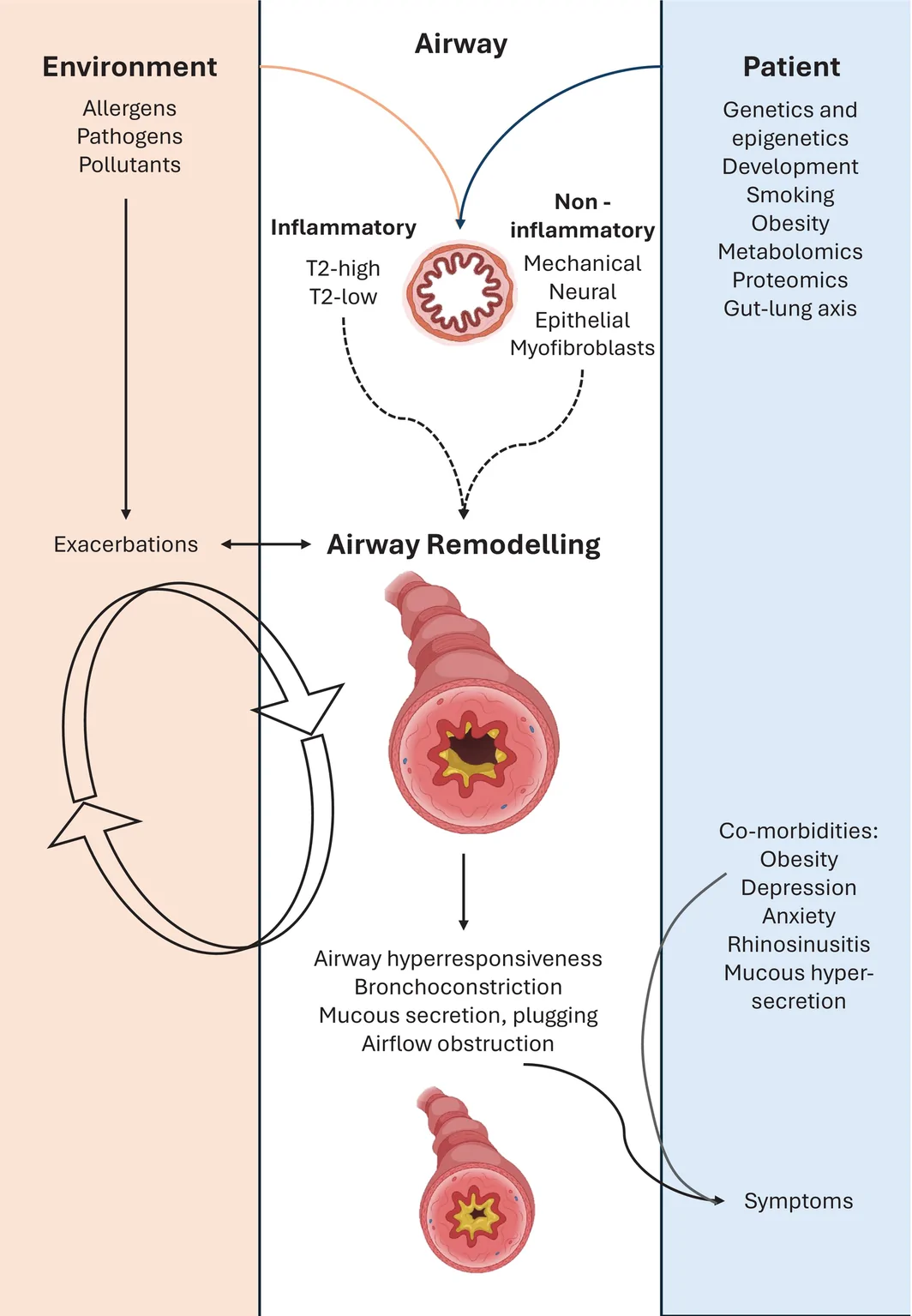
Airway remodelling is central to the pathobiology and clinical presentation of asthma. Created in BioRender. Fernando, M. (2026) https://BioRender.com/gnvsu94.

The clinical manifestations of remodelling and AHR are intermittent bronchoconstriction (excessive spontaneous or triggered day‐to‐day variability) and incompletely reversible spirometric airflow obstruction in some patients. The fact that ASM may be increased at pre‐school age before asthma onset [12], that there may be inflammation of varying types [13] or even no inflammation at all, suggest that remodelling of lung tissue and airways should be thought of and examined independently of inflammation. The latter clearly influences the clinical expression of asthma, and the clinical benefits of looking at asthma in relation to inflammatory phenotypes have proven their importance.

Endotypes and phenotypes can only be determined by our current limited knowledge of underlying mechanistic causes of asthma, and by the limited tools with which we can characterise asthma patients. Currently we could describe patients in three general domains: (1) symptoms, exacerbation and comorbidity burden (clinical), (2) irreversible airflow obstruction, excessive lung function variability, persistent moderate or severe AHR and small airway dysfunction (structural/physiological) and (3) inflammatory endotype. Each of these three domains has clinical significance in asthma and when taken together, we can treat patients more appropriately. We very much need better measurement tools, whether they be simple biomarkers or a better way of characterising lung and airway pathophysiology. These clinical factors will be considered in this review.

## 3 Clinical Phenotyping: Symptoms, Exacerbations and Comorbidities

### 3.1 Symptoms and Comorbidities

Symptoms and exacerbations are the most obvious outward manifestation of asthma that relates to the burden on the patient, family and carers. Both impact quality of life and relate to absenteeism, whilst exacerbations are strong predictors of excessive FEV_1_ decline [14]. Not all asthma symptoms or asthma‐like symptoms are due to intermittent airflow obstruction, and yet the most common pathway to diagnosis and severity is limited to symptoms only. This also applies to exacerbations which are assessed clinically.

Comorbidities influence symptoms and outcomes, particularly rhinosinusitis, obesity and psychiatric conditions, which in addition to the well‐recognised risk factors of greater treatment requirements, spirometric obstruction and previous severe exacerbations, contribute independently to an overall risk score [15]. Relatively common diagnoses that are missed include laryngeal hypersensitivity cough, inducible laryngeal obstruction, breathing pattern disorder and rhinosinusitis. At least half of patients referred to severe asthma clinics have treatment responsive asthma, that is, not severe, and improve by addressing adherence [16], inhaler technique [17], or comorbidities [18]. Therefore, characterising the nature of day‐to‐day symptoms and exacerbations thoroughly is important. Perhaps we can call this ‘symptom phenotyping’.

### 3.2 Mucus Hypersecretion

Mucus hypersecretion is an important clinical entity that affects a subgroup of asthmatics and may also be important pathophysiologically. It is more common in T2 high asthma and may relate to eosinophil peroxidase creating firm mucus gels with subsequent mucus plugging [19]. It occurs in up to 54%–63% of patients with asthma [20] and is associated with greater lung function decline, asthma symptoms and exacerbations [21]. Interestingly, patients with mucus plugging do not reliably have worse cough or sputum production than those without sputum plugging, but are more likely to have fixed airflow limitation. Whilst mucus plugging is generally stable longitudinally, any changes correspond to longitudinal changes in lung function, confirming mucus plugging as a mechanism for airflow limitation and reinforcing its clinical significance [21]. Mepolizumab, benralizumab, dupilumab and long acting muscarinic antagonists (LAMAs) reduce objective mucus burden [22, 23]. Whilst azithromycin reduced patient‐reported sputum production within the AMAZES trial, and macrolides and ICS generally have plausible biologic mechanisms in reducing sputum burden, the reduction of objectively measured sputum volume has not been demonstrated in clinical studies of these medications [22, 24].

The typical feature of productive cough is often attributed to asthma, particularly when there is associated wheeze and dyspnoea. However, the processes that lead to symptoms likely differ, that is, endo‐luminal obstruction versus airway smooth muscle contraction, that is, bronchoconstriction. Pathophysiological processes are likely to differ and management should be appropriately targeted. Clinical trials targeting bronchoconstriction are potentially confounded by participants with mucus hypersecretion. Therefore, for routine clinical management and for research purposes, mucus hypersecretion should be considered a distinct phenotype.

### 3.3 Perception of Airway Narrowing

Symptom perception is an often‐overlooked clinical phenotype which needs to be widely recognised. This is because of the reliance on symptoms for diagnosis and assessment of severity and exacerbations. Perception of airway narrowing can be easily assessed by questioning symptoms following either bronchial provocation or bronchodilator testing, or at the time of drops in peak expiratory flow rate (PEFR).

Both ‘hyper‐perception’ and ‘hypo‐perception’ have important clinical consequences. ‘Hyper‐perceivers’ have a greater symptom burden for any level of AHR or bronchoconstriction [25]. Poor perception is more common in males, the extremes of age and those not using inhaled corticosteroids [26]. Importantly, forced reduction of ICS reduces perception, which then reduces further if asthma control worsens to the point of exacerbation [27]. The risk is therefore that patients would recognise worsening later and not enact asthma action plans early enough. Poor perception is thus associated with greater risk of severe attacks [28]. In the era of ICS/formoterol PRN and maintenance and reliever therapy, it is important to recognise that patient perception of airway narrowing influences total dose, frequency and appropriateness of this therapeutic strategy [26].

### 3.4 Obesity as an Important Comorbidity

Obesity is another very important comorbidity because of its effects on airway mechanics, inflammatory pathways and symptom perception. Given these complex interactions, it is a distinctive phenotype. Cluster analyses identify obese, T2‐low patients with a high burden of symptoms and poor asthma control. Obesity reduces airway calibre and increases airway closure [29] due to reduced operating lung volume. This is a likely explanation for the high prevalence of wheeze and asthma without an increase in AHR in obese patients [30]. The association between obesity and AHR is very weak and demonstrable in men and not in women [31]. Perception of bronchoconstriction may be increased in obese asthmatics, in part due to greater expiratory flow limitation occurring for the same decrement in FEV_1_ [32], which increases the elastic work of breathing.

Asthma in obesity is generally more neutrophilic [33] and less eosinophilic, with BMI being inversely related to eosinophilic inflammation [34]. There is a skew towards Th1, Th17 and innate lymphoid cells Type 3 (ILC3) pathways producing higher levels of IL‐6, IL‐17 and IL‐22, which may diminish glucocorticoid response [35]. Importantly, obesity is identified as a significant barrier to achieving asthma remission with biologic therapy [36], possibly due to this altered symptom‐physiology interaction or lower T2‐inflammation tendency.

We propose that bariatric surgery improves asthma control and AHR primarily through reversal of mechanical stressors on airway smooth muscle including from tidal breathing at low FRC, rather than via reduction in airway inflammation [37]. Conversely, GLP‐1 receptors are expressed in lung epithelial cells, and agonists have demonstrated in vitro evidence of reduced airway inflammation, mucus secretion and AHR [38]. A retrospective cohort study demonstrated that diabetic patients with asthma receiving GLP‐1 receptor agonists had significantly fewer exacerbations and better asthma control than those on other oral anti‐hyperglycaemic agents, independent of BMI change, suggesting a weight‐independent pulmonary mechanism [38].

## 4 Structural and Physiologic Phenotypes

### 4.1 Airway and Lung Remodelling and Fixed Airflow Obstruction

Remodelling, or changes to the lung and airways that underlie asthma, should be incorporated into the conceptual framework of phenotypes for many reasons. It confirms the importance of maximising lung function and monitoring for lung function loss, it improves with treatment but requires many months to years of persistence, it is complex and varies between individuals, and a better understanding of mechanisms of remodelling is important over the long term to achieve prevention and reversal.

Remodelling of airways involves sub‐epithelial fibrosis, smooth muscle hypertrophy and hyperplasia, goblet cell hyperplasia and angiogenesis, which are independent of inflammatory phenotype [13, 39]. However, granulocytic inflammation might lead to qualitative differences in remodelling of the airway wall, that is, it may modify the processes [13]. One possible driver of remodelling is mechanical stress of the airway wall due to bronchospasm, which stimulates epithelial release of pro‐remodelling mediators including fibronectin, MMP‐9 and collagen III and IV [40]. Epithelial—mesenchymal transition (EMT) where epithelial cells change to a mesenchymal phenotype could also promote remodelling, but from non‐allergic stimulation of the airway epithelium [41]. Airway smooth muscle is also likely to drive remodelling. It is functionally plastic, being able to rapidly adapt to maximise contractile function in response to stretch or shortening [42], and can also transition from a contractile phenotype to a proliferative phenotype [43], secreting pro‐remodelling and pro‐inflammatory cytokines. Pro‐remodelling mediators such as TGF‐β, EGF and MMPs are also produced by senescent airway smooth muscle cells, epithelial cells and fibroblasts, which develop a senescence‐associated secretory phenotype in response to various cellular stressors that accumulate with aging [44].

In T2‐high asthma, many components of remodelling including reticular basement membrane thickness, airway smooth muscle mass, epithelial damage, proliferation markers and extracellular matrix are improved by ICS [45] and anti‐IL‐5 biologics [46, 47]. Notably, tezepelumab did not significantly alter reticular basement membrane thickness or epithelial integrity despite improved AHR [48].

Fixed airflow obstruction is a phenotype that affects 35%–50% of asthmatics with strong evidence that not only the airways, but abnormalities of the lung are also fundamental to this defect [49]. Disruption of terminal bronchiolar‐alveolar attachments, alveolar dilatation and loss of elastic recoil in asthma reduce distending forces on the airways which are critical for preventing airway closure and narrowing [50]. Risk factors are longer asthma duration, male sex, higher smoking rates and uncontrolled T2‐high inflammation, with small airways being the dominant site of obstruction [51]. CT airway measurement correlates with histological features of remodelling [52], but is limited by the spatial heterogeneity of the remodelling process, confounding effects of oedema and intraluminal mucus on wall parameters, and insufficient resolution to distinguish discrete wall layer components. Given the above complexity of remodelling, progress in understanding its pathogenesis will require better tools, such as polarisation sensitive optical coherence tomography, which can image the full thickness of small airways and resolve ASM [53], combined with cellular and molecular studies and comprehensive clinical phenotyping.

### 4.2 AHR And Day‐To‐Day Variability

Increased day‐to‐day variability of lung function and AHR are fundamental to asthma and therefore need to be part of clinical phenotyping. Mechanisms driving AHR are poorly understood, but include changes in ASM structure and contractility, airway remodelling, airway inflammation and neural remodelling [54, 55]. Neural remodelling involves the expansion of airway sensory nerves, together with functional changes in sensory and autonomic pathways, and interacts bidirectionally with immune cells and structural airway cells responsible for inflammation and remodelling. Inflammatory mediators can sensitise sensory nerves and increase TRPV1 expression or activity, while activation of TRPV1‐expressing afferents promotes neuropeptide release, mucus secretion, neurogenic inflammation, cough hypersensitivity and airway hyperresponsiveness [55].

AHR is a predictor of future risk including lung function decline even in those with or without asthma [54], persistence of asthma from childhood to adulthood [56], exacerbation risk [57] and loss of control with ICS downtitration [27]. Although symptoms and morning PEFR improve within weeks of initiating ICS treatment, which is probably due to reduction in inflammation, AHR takes months to years to improve or to resolve [58]. The slower time course of improvement in AHR parallels the time course for improvements in biopsy markers of remodelling [45].

Increased day‐to‐day variability in lung function is the cornerstone of the definition of asthma and what causes asthma symptoms. However, airway narrowing may only vary slowly over days, for example, with increasing inflammation associated with viral infection leading to inflammation and mucus plugging. Despite this, day‐to‐day monitoring of lung function is not the norm but the exception, even at diagnosis. This leads to both over and under‐diagnosis of asthma.

There are challenges to peak flow monitoring, including unfamiliarity with interpreting PEFR diaries, educating patients on the importance and utility, and adherence. However, our experience is that it is critical to diagnosis, severity assessment, and management of exacerbations, especially in severe disease. Patients appreciate having an objective marker of asthma activity. Although the few published studies do not show improved outcomes in relation to peak flow versus symptom based plans, they were underpowered and used ineffective asthma action plans, and were combined with other interventions such as regular follow‐up [59, 60]. A seminal study showed that quadrupling ICS dose in action plans reduced oral corticosteroid use for exacerbations by 20%, despite suboptimal adherence to plans [61].

We also suggest that PEFR interpretation needs to be quick and simple. Published data in healthy subjects used impractical methods to calculate variability, including calculating differences between consecutive days and between morning and afternoon [62]. The simplest method is taking the best recorded PEFR during a stable period, and comparing this to the worst value in a clinically relevant period, for example, 1 month. For example a lowest PEFR of 400 L/min in comparison with a recent best of 500 L/min equates to (500 − 400)/500 × 100% = 20% variability, and referred to as ‘percent of recent best’ [63]. Surprisingly, there are no published data from healthy subjects in which variability was calculated in this manner. Such studies would be very useful.

Newer technology will ultimately replace mechanical peak flow metres and paper diaries. Respiratory oscillometry shows promise and has the added advantage of requiring only relaxed tidal breathing during measurement and is thus easy to self‐administer [64]. Data are electronically stored automatically and can be transmitted remotely, which allows automated analyses. Parameters that have been developed to predict exacerbation onset still need prospective evaluation [64].

### 4.3 The Small Airways

Although the underlying mechanisms of AHR are complex and poorly understood, an important site is the small airways, given the strong association between AHR and small airway dysfunction measured by multiple breath nitrogen washout (MBNW) [65]. Small airway dysfunction is common, particularly in severe asthma. In the ATLANTIS multi‐centre, multi‐national study of patients from the general population and from general and specialist practice [66], the prevalence of small airway dysfunction was dependent on the measurement method, that is, mid‐expiratory flows, oscillometry, multiple breath nitrogen washout (MBNW) or gas‐trapping on plethysmography. MBNW and oscillometry are established lung function measurements of small airway function, which show around 20% and 40% prevalences of abnormality, respectively. Small airway dysfunction is associated with poor asthma control and more frequent exacerbations, supporting its use to complement spirometry [66, 67, 68, 69]. Abnormal acinar ventilation is associated with risk of asthma related hospital admission [67] and loss of control with ICS withdrawal [68]. Abnormal oscillometric reactance (a sensitive measure of small airway dysfunction) relates better to asthma control than does spirometry [69] and during monitoring in a clinic, changes in oscillometry and spirometry were concordant [70].

Small airway dysfunction can be targeted with fine‐particle inhaled corticosteroids, which have greater deposition in small airways compared with larger particles and are associated with improved oscillometry parameters [71]. Small airway function improves following dupilumab and tezepelumab, which may be because the injected agent can reach severely diseased small airways [66, 72].

Small airway dysfunction is also measurable using functional imaging, such as ventilation Single Photon Emission Computed Tomography using Technegas as a radioactive marker, or by magnetic resonance (MR) using hyperpolarised He3 or Xe [73]. Three‐dimensional imaging provides topographic information on extent and location of the most dysfunctional airways, which relates to lung function measures. Ventilation measured by hyperpolarised Xe MR demonstrates improvements with dupilumab [7] and single inhaler ICS/LABA/LAMA treatment [23]. Therefore, information from 3‐dimensional ventilation imaging is needed in future mechanistic studies, particularly if combined with cellular and ultra‐structural information.

## 5 Type 2 Inflammation (T2) High Endotype

### 5.1 Genetics

The prevalence of T2‐high asthma ranges from 27% in the general population of patients with asthma to 55% in a study of patients visiting a severe asthma clinic [74, 75]. Heritability of allergic asthma ranges between 35% and 95% and concordance in monozygotic twins is between 60% and 80%, which is several times higher than that in dizygotic twins [76]. Single nucleotide polymorphism variants at gene loci responsible for components of the T2 inflammatory pathway consistently appear in genome‐wide association studies, including TSLP, IL‐13, IL‐33 and the master transcription factor for Th2 cells, GATA3 [77]. Despite ongoing identification of novel gene associations, these still do not explain the entirety of asthma susceptibility. Epigenome‐wide association studies (EWAS) partially address this gap, identifying thousands of implicated, differentially methylated cytosine‐phosphate‐guanine (CpG) nucleotides [77]. Distinct DNA methylation profiles are linked with T2‐high inflammatory biomarkers and may offer a novel method of detailed endotype characterisation [78].

### 5.2 Immunopathology

Disruption of airway epithelial barrier integrity (reduced e‐cadherin and claudin‐18) occurs in asthma, is partially restored by inhaled corticosteroids [40], and is worsened by a variety of insults, for example, proteolytic allergens including house dust mite and *Alternaria* fungi, and by cytotoxic viral pathogens [33, 76]. This allows other pollutants, viruses, bacteria and allergens to enter and cause inflammation. Once the epithelium is breached, antigen‐presenting cells display allergens to naïve CD4+ T cells, where GATA3 induction facilitates differentiation into Th2 cells [33]. Alarmin release (IL‐33, IL‐25 and TSLP) activates innate immune cells including mast cells, basophils and innate lymphoid cells Type 2 (ILC2), which favours Th2 polarisation [76]. However, why this might affect some individuals and not others is not known. Not all atopic individuals develop asthma, despite airway inflammation in viral infection being common and practically universal.

The downstream effects of T2 cytokines are familiar to many clinicians, driving IgE production, goblet cell growth and mucus secretion, eosinophil migration and activation, exhaled nitric oxide (eNO) release and increased ASM contraction.

### 5.3 The Gut‐Lung Axis

Dysbiosis within the gut microbiome can modulate T2‐high airway inflammation. Intestinal bacteria ferment dietary fibres into short chain fatty acids (acetate, propionate, butyrate) and convert tryptophan into various metabolites, which collectively promote bronchial epithelial barrier function whilst suppressing ILC2 and Th2 driven airway inflammation and airway hyperresponsiveness [79]. However, the relative impact of dysbiosis on T2‐inflammation in asthma has not been clearly quantified, and a clear clinical benefit of dietary fibre supplementation or probiotics has not been demonstrated in adults.

### 5.4 Allergic Asthma

The allergic endotype is universally considered but is of limited clinical utility. The association between allergy and asthma does not infer causality. The evidence to support the often‐quoted cause of asthma: allergic sensitisation → T2 high inflammation in predisposed individuals → airway remodelling → clinical asthma, is poor. The IgE level in allergic asthma does not consistently relate to asthma exacerbations or other clinical outcomes and it is not predictive of clinical response to current biologics [80].

Knowledge of the allergic asthma phenotype is helpful in specific clinical situations; (1) patients are generally interested in knowing allergic status and often blame allergic sensitisation (wrongly) as a cause for asthma, (2) prescribing omalizumab or dupilumab (in Australia), (3) when considering allergen desensitisation (allergen avoidance may require extreme measures, for example, relocating to alpine environments [81] to be effective), (4) exacerbation‐prone and severe asthma, (5) informing likelihood of spontaneous remission between childhood and adulthood and (6) identifying patients at risk of ‘Thunderstorm asthma’ in regions where it has occurred.

Subcutaneous and sublingual allergen therapy reduces ICS requirement in non‐severe allergic asthma while studies in severe asthma are limited due to the potential for triggering severe asthma attacks, particularly in patients with reduced FEV_1_ [82]. The addition of omalizumab in conjunction with allergen desensitisation may mitigate this risk and improve asthma outcomes in children with moderate to severe allergic asthma [83].

*Alternaria* sensitisation is independently associated with clinical severity indicators, including respiratory arrest and death [84], and mono‐sensitisation is associated with poorer symptom control, worse airflow obstruction, more exacerbations, and older age of onset [85]. Mono‐sensitisation to *Alternaria* mould might therefore raise the possibility of allergen avoidance in severe asthma, even though there is little evidence of efficacy, particularly in mould allergen avoidance [86]. *Alternaria* grows on rotting vegetation and allergen release in the form of spores is related to changes in environmental conditions. Mono‐sensitisation provides circumstantial evidence that *Alternaria* sensitisation and exposure contribute to severity, but establishing the link is complicated by difficulties in obtaining exposure data. Sensitisation to other allergens and polysensitisation are not related to asthma severity [87].

Early childhood onset allergic asthma is the ‘classic’ or ‘archetypic’ endotype which drove the allergic paradigm of asthma. Typical features include childhood onset with allergic rhinitis and eczema, and eosinophilic airway inflammation [88]. Childhood‐onset allergic asthma remits in 26% based on clinical criteria and 15% based on the absence of bronchial hyperresponsiveness [89]. Risk factors for persistence include allergic sensitisation, female sex, greater severity, AHR and impaired lung function [56, 89]. Understanding the mechanisms of remission between childhood and adulthood might provide treatable targets.

‘Thunderstorm’ asthma is specific to certain antigens. The event in Melbourne, Australia in November 2016, due to a sudden large mass of fine‐particulate rye‐grass pollen being dumped on the city, led to 3365 excess respiratory presentations to ED and 476 excess asthma admissions, with 28% having a previous diagnosis of asthma. Deaths occurred in those with known asthma, with people of Asian and Indian heritage over‐represented among fatal cases. Similar epidemic events have occurred with *Alternaria* fungi (Europe), olive tree pollen (Italy) and *Artemisia* pollen (China) [90].

### 5.5 Eosinophilic Asthma

The eosinophilic endotype is arguably the easiest to manage in terms of pathways, from patients who have minimal symptoms, AHR, PEF variability and spirometric impairment, through to those with severe derangement of those four clinical indicators. This is because blood eosinophilia, as a surrogate of eosinophilic airway inflammation, performs very well in predicting response to corticosteroid and biologic therapies [91]. Despite this, blood eosinophils are not a highly sensitive marker of airway eosinophilia and longitudinal measurements are required to detect occult T2 airway inflammation [92]. Although blood eosinophil counts vary over time [93] and diurnally [82], are reduced by oral corticosteroids by around 80% [94] and by ICS by around 20% [95], they are highly clinically significant in asthma because of their indication of risk. Blood eosinophilia is associated with more frequent exacerbations, poor asthma control and longitudinal lung function decline [92].

### 5.6 FeNO High Asthma

FeNO is a non‐invasive biomarker of T2‐high inflammation driven by both IL‐4 and IL‐13 stimulation of inducible nitric oxide synthase in bronchial epithelial cells [96]. As eosinophils are primarily driven by IL‐5, FeNO reflects a distinct component of T2 inflammation; the combination of elevated FeNO and eosinophilia are synergistic risk factors for exacerbations [97]. Similar to eosinophils, elevated FeNO is independently associated with poor asthma control, impaired lung function, response to ICS and lung function decline [96]. Specifically, elevated FeNO predicts accelerated lung function decline both in patients with controlled asthma and those with more severe asthma [96, 98]. High FeNO may therefore influence treatment escalation to preserve lung function. The great limitation is that the understanding of mechanisms for accelerated lung function decline is incomplete and evidence that current treatments are effective for this is poor. In individuals who qualify for both anti‐IL‐4/13 and anti‐IL5 treatment, high FeNO with only modestly elevated blood eosinophils, that is, around 300/μL, might push the decision towards dupilumab or tezepelumab, given that FeNO predicts clinical response [91, 96].

As with other biomarkers in asthma, care should be taken when incorporating FeNO measurements into clinical practice, because of the limitations in measurement. The Global Lung Function Initiative determined that the high degree of variation in values between sites and devices precluded development of predicted values in healthy subjects [99]. Also relevant to interpretation is the effect of diurnal variability, rhinosinusitis, allergen exposure, smoking, nitrate rich diets and acute bronchoconstriction on measured FeNO levels [82]. However, with compatible clinical features, values > 50 ppb can support a diagnosis of T2‐high asthma [82]. The failure of suppression of FeNO may also be utilised to determine treatment adherence [82].

### 5.7 Non‐Allergic Type 2 High Asthma

The non‐allergic pathway to eosinophilic airway inflammation has similarities with the allergic pathway, in that environmental insults on the airway epithelium rather than allergic reactions lead to TSLP secretion which stimulates ILC2 cells (rather than Th2 cells) to produce IL‐5 and IL‐13 resulting in eosinophil recruitment, mucus secretion and smooth muscle contractility [33, 92]. Nitric oxide production and eosinophilia can be more prominent and persistent than in the allergic eosinophilic endotype [100]. ILC2 cells have been quantified in sputum of asthma patients and have high specificity for eosinophilic airway inflammation [101].

Patients in this endotype typically present in adulthood, account for 21%–48% of patients with severe asthma [102] and have also been identified in several cluster analysis studies [88, 103]. There is a strong association with chronic rhinosinusitis with nasal polyposis. Patients often have fixed airflow limitation, poor asthma control, and frequent exacerbations with resistance to corticosteroids [92].

## 6 T2‐Low Asthma

It is curious to us that this endotype is known by something that it *is not*, rather than by the pathophysiological pathways that are present. There is uncertainty about what the biomarkers actually represent, that is, low FeNO and eosinophil levels could be due to a marked corticosteroid response, or that the underlying causes and drivers of activity are driven by processes where T2 inflammation is completely absent [33, 35]. From a clinical perspective, it reinforces the importance of undertaking a comprehensive assessment before embarking on treatment, rather than treating any suspected asthma with ICS ± LABA based on symptoms alone, which is the norm in general as well as specialist practice. Nevertheless, the practical clinical problem is still how to manage uncontrolled symptoms, lung function impairment, ongoing bronchoconstriction, exacerbations and whether or not T2 inflammation was suppressed, or never there at all.

Patients are more likely to have adult‐onset asthma, be female, obese, and have a significant smoking history. Symptom burden can be high with less bronchodilator responsiveness and poor response to corticosteroids [35]. This phenotype has emerged consistently in multiple cluster analyses [88, 103].

The origins of airway remodelling in T2 low endotypes have been discussed above where remodelling may be initiated by epithelial insult but then driven by myofibroblasts and ASM with ongoing stimuli, such as mechanical stress, neural mechanisms or ECM changes. Irritants and insults may include viral infections, bacterial colonisation including *Haemophilus influenzae* , fungal sensitisation, cigarette smoke or environmental pollutants [76].

T2‐low asthma can exhibit sputum neutrophilia (sputum neutrophils > 60%) or neither sputum eosinophilia nor neutrophilia (paucigranulocytic) [33]. In neutrophilic asthma, gene expression of inflammasome‐related components such as Nod‐like receptors (NLR), caspases and IL‐1β is elevated. This results in increased IL‐1 production, Th17 cell development and airway neutrophil recruitment. Macrophage migration inhibitory factor (MIF) augments inflammasome assembly and diminishes response to glucocorticoids [35]. Th17 skewed inflammation is driven by IL‐1, IL‐6 and TGF‐β, producing IL‐17, IL‐22, IL‐8 and CXCL neutrophil attractants. Th1‐mediated cytokines may further amplify this through production of IFN‐γ and TNF‐α [33, 76]. These pathways may be modulated by long, non‐coding RNAs (lncRNAs) or microRNAs, which can impact Th17/Treg cell imbalance, Th1/Th2 activity imbalance, macrophage polarisation and glucocorticoid insensitivity [104]. For example, sputum microRNA profiling has identified neutrophil‐derived *hsa‐miR‐223‐3p* as a regulator of Th17 and inflammasome pathways in neutrophilic asthma [105]. Given the lack of specific drug treatments for T2‐low asthma, particular attention to comorbidities is essential, especially mucus hypersecretion, obesity, smoking and other environmental exposures.

Trained immunity within airway immune cells can modulate pro‐inflammatory and anti‐inflammatory pathways across endotypes, particularly in T2‐low mechanisms. Airway macrophages, neutrophils and epithelial cells can undergo intrinsic changes following an initial stimulus, resulting in a long‐lasting immune memory analogous to that of the adaptive immune system. Importantly, inciting stimuli are not limited to allergens, and include viruses, bacteria or pollutants. Initial exposure causes epigenetic reprogramming via histone modifications or DNA methylation which can enhance the transcription of pro‐inflammatory genes for cytokines such as TNF‐α and IL‐6 [106]. This combined with enhanced activity of intra‐cellular glycolytic metabolic pathways compared to oxidative mechanisms supports augmented and sustained inflammatory responses upon repeated stimulus [106], which may be a contributory mechanism for non‐allergic asthma exacerbations and T2‐low inflammation more broadly. Aside from preventing initial and repeated stimulus through vaccination, smoking cessation or pollutant avoidance, further evidence is needed to inform specific targeting of trained immune pathways in asthma.

Macrolides exert broad anti‐bacterial, anti‐viral, anti‐inflammatory and immunomodulatory effects. In T2‐low asthma, key mechanisms include reduction in NFkB, IL‐17, TNF‐α and PI3K inhibition, limiting neutrophil activity and potentially ameliorating corticosteroid resistance [107]. The AMAZES trial demonstrated significant reductions in exacerbations and respiratory infections with azithromycin in both eosinophilic and non‐eosinophilic sub‐groups, with improved quality of life scores. Clarithromycin reduced airway IL‐8 and MMP‐9 and improved quality of life in neutrophilic asthma [107]. These benefits must be weighed against risks of antibiotic resistance, non‐tuberculous mycobacterial resistance, QTc prolongation and gastrointestinal side‐effects.

Tezepelumab reduces exacerbation rate in T2‐low asthma, albeit providing greater benefit in T2‐high asthma. A plausible mechanism is through TSLP receptor‐mediated effects on mast cells and airway hyperresponsiveness, which is distinct from its T2 anti‐inflammatory actions [10, 91]. Astegolimab (anti‐ST2/IL‐33) reduces exacerbation rates and improves FEV_1_ in eosinophil‐low patients [108]. Conversely, itepekimab (also anti‐IL‐33) did not demonstrate clear evidence of improved outcomes within a small subset of included T2‐low patients [109]. The tyrosine kinase inhibitor imatinib improves AHR, mast cell count, and tryptase levels in patients with severe asthma (unselected by endotype), though greater improvements in AHR were seen in those with lower blood eosinophils [110]. Suppressing mast cell activity may therefore be a useful target in T2‐low asthma, though studies of other tyrosine kinase inhibitors have not been performed in this subgroup specifically [107]. Several T2‐low targeted therapies including CXCR2 antagonists, anti‐TNF, anti‐IL‐17 and anti‐IL‐23 agents have failed to demonstrate clinical benefit or even caused harm [35, 107]. These mixed findings suggest that T2‐low patients are either not being precisely selected in studies or may be a heterogenous group of patients without a unifying mechanism, requiring further mechanistic understanding or reclassification altogether. Seeking mechanisms beyond the effector cells of inflammation may enable a more accurate sub‐classification of the T2‐low group and asthma in general, with multi‐omic methods offering potential to achieve this.

## 7 Emerging Role of Multi‐Omics

### 7.1 Multi‐Omic Endotype Characterisation

Multi‐omic analysis may ‘sub‐endotype’ patients beyond inflammatory characteristics alone and capture genomic, proteomic, metabolomic and microbiome contributions to asthma pathophysiology and treatment efficacy. Analysis of volatile organic compounds (VOCs) in breath (breathomics), urine or blood can effectively differentiate patients with asthma, as well as characterise those with eosinophilic or non‐eosinophilic airway inflammation [111]. Multi‐omic based cluster analyses from sputum in patients with asthma can identify more detailed asthma sub‐types within traditional cytology‐defined endotypes, for example, eosinophilic or neutrophilic [112]. Metabolomic studies suggest that altered levels of bile acids and sphingolipids are associated with asthma and its severity [111], whilst urinary carnitine metabolism signatures are strongly associated with asthma severity and relatively unaffected by oral corticosteroid treatment, suggesting a potential role as a biomarker or treatment target in severe asthma [113].

### 7.2 Multi‐Omic Prediction of Biologic Response

Machine‐learning derived IL‐21 related genetic risk scores were able to predict clinical response (> 50% reduction in exacerbations) to omalizumab weakly (area under ROC curve of 0.62) and dupilumab (area under ROC curve of 0.76), though this was limited by a lack of comparison to baseline T2 inflammation biomarker levels [114]. Integration of metabolomic and proteomic data in patients treated with mepolizumab (but not omalizumab) identified distinct profiles which changed following treatment and correlated with clinical improvements [115]. Whilst an abundance of multi‐omic approaches exist, clear evidence demonstrating a clinical benefit in using these to sub‐endotype patients and to inform treatment is still needed to guide their use in future clinical practice.

## 8 Conclusion

Asthma management is undergoing a paradigm shift from stepwise, symptom‐driven therapy towards personalised, mechanism‐based care. The endotype‐phenotype framework provides a practical structure for this transition. Major challenges that currently exist are using existing tools better, that is, peak flow monitoring, bronchial challenge testing, estimation of perception of bronchoconstriction and having better tools for the future, for example, oscillometry as a measure of small airway dysfunction and imaging, that is, ventilation and airway structural imaging.

The T2‐low endotype is likely a mechanistically heterogenous group requiring more detailed pathophysiologic assessment and classification, which multi‐omic approaches may be able to facilitate in future with progression of clinically focused research. Existing drugs may be useful in specific phenotypes, for example, GLP‐1R agonists in obese asthmatics, regardless of inflammatory phenotype. One of the biggest challenges is improving remodelling, which has seen little progress. The need for better tools is critical since bronchial biopsy on its own is insufficient and impractical for mechanistic studies or endotyping. In the meantime, clinicians and researchers can do better by comprehensively assessing patients and participants, based on an in‐depth understanding of pathophysiology and hence of each patient's endo‐phenotypic profile. A summary of this approach is presented in Table 1, with a focus on several, non‐exhaustive endo‐phenotypes that may be observed alongside, or irrespective of T2 inflammatory endotype.

**TABLE 1:** Measurable, prognostic and treatable endophenotypes that may be observed in relation to, or irrespective of inflammatory endotype.

Endo‐phenotype	Measurement	Risk	Targeted treatment options
Mucous hyper‐secretion and plugging	CT imaging	Exacerbations ↑	Dupilumab, benralizumab, tezepelumab, macrolide, LAMA, inhaled corticosteroid
		Asthma control ↓	
		Lung function decline	
Rye grass pollen sensitisation	Specific‐IgE	Thunderstorm asthma: exacerbations ↑, hospitalisations ↑, mortality ↑	Regular inhaled corticosteroid, evaluation for asthma in sensitised patients with allergic rhinitis, avoidance, desensitisation
	Skin prick testing		
*Alternaria* sensitisation	Specific‐IgE	Severe asthma, thunderstorm asthma? ↑mortality	Avoidance, desensitisation
	Skin prick testing		
Airway remodelling	CT imaging, complex lung function, PS‐OCT	Lung function decline, exacerbations ↑	Treat underlying inflammatory and non‐inflammatory causes.
			In T2‐high asthma: ICS, mepolizumab, benralizumab, omalizumab, dupilumab.
Airway hyper‐responsiveness	Bronchoprovocation testing	Lung function decline, exacerbations ↑	ICS, tezepelumab
	Peak flow variability		
	Ventilation heterogeneity: SPECT–CT (V/Q), PET, multi‐breath nitrogen washout		
Poor perception of airway narrowing	Perception of peak flow variability, bronchodilator response, interval lung function declines or bronchoprovocation	Exacerbations ↑, mortality ↑	Objective airway narrowing monitoring, peak flow diaries and peak flow based asthma action plan
Fixed airflow limitation	Pulmonary function testing	Exacerbations ↑	Early ICS/LABA/LAMA, biologics for T2‐inflammation related remodelling
Small airways dysfunction	Oscillometry, multi‐breath nitrogen washout	Exacerbations ↑, asthma control ↓	Fine‐particle inhaled corticosteroids, dupilumab, tezepelumab

## Author Contributions

**Mario Fernando:** conceptualization, writing – original draft, writing – review and editing, visualization, data curation. **Gregory G. King:** conceptualization, data curation, supervision, writing – original draft, writing – review and editing, visualization.

## Funding

This work was supported by the National Health and Medical Research Council, 2050302.

## Conflicts of Interest

G.G.K. is a non‐executive board member of CycloPharm, Australia, and of the Thoracic Society of Australia and New Zealand, and has research received grants from Chiesi Pharma, Australia, and CycloPharm, and fees for consultancies and presentations from AstraZeneca, Chiesi Pharma, GSK and Sanofi. M.F. declares no conflicts of interest.

## Data Availability

Data sharing not applicable to this article as no datasets were generated or analysed during the current study.
